# Handling rescue therapy in myasthenia gravis clinical trials: why it matters and why you should care

**DOI:** 10.1002/acn3.52309

**Published:** 2025-04-16

**Authors:** Justin M. Leach, Inmaculada Aban, Gary Cutter, Michael Benatar

**Affiliations:** ^1^ Department of Biostatistics University of Alabama at Birmingham Birmingham Alabama USA; ^2^ Department of Neurology University of Miami Miller School of Medicine Miami Florida USA

## Abstract

Myasthenia gravis (MG) clinical trials typically allow rescue therapy during follow‐up in the event of marked worsening of MG symptoms. Failure to appropriately address rescue therapy in defining treatment effects and planning statistical analyses may yield biased estimates, increase false positive rates, or decrease statistical power – all of which increase the risk that inaccurate information influences patient care. In alignment with recent International Council for Harmonisation of Technical Requirements for Pharmaceuticals for Human Use (ICH) guidelines, we review strategies based on the estimand framework that produce rigorously defined treatment effects in MG trials where rescue therapy may be administered. We also discuss the interpretation, clinical relevance, and pitfalls of each strategy in the context of MG trials, suggesting circumstances in which a strategy would or would not be appropriate. Finally, we outline available statistical methods for estimating treatment effects based on each strategy. As primary endpoints and statistical analyses for trials must be defined prior to conducting the study, it is necessary to consider how to address rescue therapy during study planning.

## Introduction

Autoimmune myasthenia gravis (MG) is a disorder of neuromuscular transmission that leads to fatigable weakness of skeletal muscles, manifesting with weakness of extraocular, bulbar, neck, respiratory, and limb muscles.^1^, ^2^ Correspondingly, MG outcome measures aim to improve how patients feel and function. Several measures have been developed to quantify daily functioning and/or quality of life (QOL), including the MG activities of daily living (MG‐ADL), the quantitative MG (QMG) score, the MG‐QoL15r, the MG Composite, and the MG Impairment Index (MG‐II), with the MG‐ADL and QMG most commonly used as outcome measures in clinical trials.^3^, ^4^, ^5^, ^6^, ^7^ Endpoints in MG trials are usually defined as changes in the chosen measure between baseline and a prespecified follow‐up, typically 6–26 weeks (Table 1).

**Table 1:** MG trial approach to handling rescue therapy.

Author (year)	Trial name	Phase	Treatment versus comparator	Sample size	No. (%) rescue therapy	Duration	Statistical method	Strategy^a^
Antozzi (2024)	–	2	Nipocalimab (four dose levels) versus placebo	68	2 (2.9%)	57 days	Mixed model for repeated measures including baseline covariates	Hypothetical
Bril (2021)	–	2	Rozanolixizumab versus placebo	43	0^b^	29 days	Linear mixed model(s) including baseline covariates	Unknown^c^
Bril (2023)	MycarinG	3	Rozanolixizumab (two dose levels) versus placebo	200	3 (1.5%)	6 weeks	Values at‐ and postrescue therapy set to “missing.” Maximum likelihood under missing‐at‐random assumption	Hypothetical
GomezMancilla (2024)	–	2	Iscalimab versus placebo	44	5 (11.4%)	25/52 weeks	Linear mixed model(s) including baseline covariates	Unknown^c^
Howard (2017)	REGAIN	3	Eculizumab versus placebo	125	18 (14.4%)	26 weeks	Worst‐rank ANCOVA with rankings based on time‐to‐rescue therapy (first to receipt is ranked last), followed by change‐from‐baseline (most improved is ranked first)	Composite
Howard (2019)	–	2	Efgartigimod versus placebo	35	1 (2.9%)	11 weeks	Mixed model for repeated measures including baseline covariates	Hypothetical
Howard (2021)	ADAPT	3	Efgartigimod versus placebo	167	3 (1.8%)	Up to 26 weeks	Multivariable logistic regression	While‐on‐treatment
Howard (2020)	–	2	Zilucoplan versus placebo	44	4 (9.1%)		ANCOVA on change‐from‐baseline with treatment as factor and baseline QMG as covariate and LOCF‐imputation for measurements postrescue therapy	Hypothetical
Howard (2023)	RAISE	3	Zilucoplan versus placebo	174	14 (8.1%)	12 weeks	Linear mixed model(s) including covariates. Postrescue therapy values were imputed as baseline or last score, whichever was worse	Hypothetical
Hewett (2018)	–	2	Belimumab versus placebo	40	0^b^	24 weeks	Mixed model for repeated measures including baseline covariates	Unknown^c^
Piehl (2022)	RINOMAX	N/S	Rituximab versus placebo	47	9 (19.1%)	16 weeks	Fisher's exact test	Composite
Sharshar (2021)	–	N/S	Slow‐ and rapid‐corticosteroid tapering	117	12 (10.3%)	15 months	Mantel–Haenszel risk difference and risk ratio stratified by group of centers and thymectomy at inclusion	Treatment policy
Wolfe (2016)	MGTX	N/S	Thymectomy + prednisone versus prednisone only	126	31 (25.2%)	3 years	2‐sample *t*‐test	Treatment policy
Yan (2024)	–	3	Batoclimab + standard of care versus placebo + standard of care	132	4 (3.03%)	24 weeks	Multivariable logistic regression	Treatment policy

Studies included based on PubMed search (date: 2024‐06‐05). Restricted to 2016–2024 using terms “myasthenia gravis” [Title] and article types “Clinical Trial” or “Randomized Controlled Trial.” Additional exclusions included complex designs, primary outcome not functional status, intervention other than medication/treatment, secondary analyses, open‐label extensions, phase 1 trials, pilot studies, animal studies, studies without comparator groups, or retracted.

N/S, not specified.

^a^ If the strategy was not explicitly stated (usually the case), then we attempted to infer the strategy based on the study protocol and statistical analyses.

^b^ Rescue therapy is not explicitly mentioned in the manuscript.

^c^ Insufficient details in the manuscript to determine how post‐rescue therapy observations were handled. Note that a mixed model for repeated measures does not necessarily indicate a hypothetical strategy with respect to rescue therapy if postrescue therapy observations are not excluded (similarly for linear mixed models and while‐on‐treatment/hypothetical strategies).

**Figure 1:**
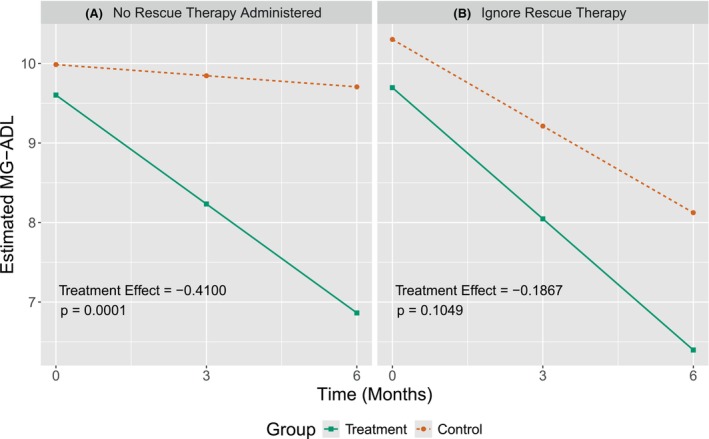
Data simulation to illustrate the impact of rescue therapy on trial results. (A) Analysis based on linear mixed model (LMM) when no patients receive rescue therapy. (B) Rescue therapy is imposed on the simulated data set between months 3 and 6; 72 (24%) total, with 21 (14%) and 51 (34%) in the treatment and control groups, respectively. Rescue therapy improved the 6‐month MG‐ADL measurement by 50%. By fitting an LMM *ignoring the use* of rescue therapy, the improvement of MG‐ADL in both control and treated patients is partly due to the rescue therapy, and the groups appear more similar due to rescue therapy, not lack of treatment efficacy.

MG trials aim to evaluate an experimental therapeutic's ability to improve MG patients’ daily functioning, but in the event of marked worsening of MG symptoms, rescue therapy with IV immunoglobulin (IVIg) or plasma exchange (PLEX), for example, is available, complicating between‐group comparisons.^8^, ^9^ Ignoring rescue therapy can, in the worst cases, lead to incorrect conclusions. Consider a situation where the population of interest is MG patients with refractory (treatment‐resistant) MG and thus exacerbations are likely. For an effective experimental treatment, we may expect the experimental treatment group to have lower rescue therapy rates than the standard‐of‐care group because those in the treatment group are more likely to have better functional outcomes and thus less likely to require additional treatment. However, patients who receive rescue therapy are likely to benefit from the rescue therapy, in which case the treatment effect estimate will be biased toward the null if we ignore rescue therapy. In this example, the standard‐of‐care group appears more similar in their endpoints to the experimental treatment group due not to the effectiveness of the standard‐of‐care but to having received rescue therapy (Fig. 1). That is, the treatment will appear *less effective* than it is (bias), making it less likely that the results of hypothesis testing will produce a statistically significant result (reduced power), thereby potentially leading us to discard an effective treatment. In such cases, appropriately accounting for rescue therapy could (1) reduce bias and (2) increase power. On the other hand, consider the example of a treatment that is ineffective and/or exacerbates disease, in which case the treatment group may have higher rates of rescue therapy than the control group. Here, ignoring rescue therapy may make the treatment group appear less similar to the control group, but on account of rescue therapy rather than the experimental treatment. That is, the treatment will appear *more effective* than it is (bias), making it more likely that the results of hypothesis testing will be significant (false positive), thereby leading us to recommend an ineffective treatment or overstate the benefits of a mildly effective treatment. In such cases, accounting for rescue therapy would (1) reduce bias and (2) decrease false positive risk. In short, ignoring or improperly accounting for rescue therapy can bias the treatment effect estimate, increase false positive risk, and decrease power, that is, increase the risk of reaching a biased or incorrect conclusion that then inappropriately informs patient care.

Randomization does not help to overcome this problem. To make causal conclusions, observed differences in endpoints between treatment groups must be due to differential effects between the experimental and control therapies, not other chance or systematic differences between groups. There are two primary ways that differences between groups bias study results: (1) baseline differences between groups on variables that can affect the outcome (confounding), for example, if at baseline one group had more severe disease on average than the other and (2) events that occur postbaseline at different rates between groups (intercurrent events), for example, rescue therapy. On average, randomization results in balance between groups across baseline characteristics that could affect the outcome, thereby avoiding systematic bias due to confounding.^8^, ^9^ Unfortunately, randomization cannot address bias arising from postrandomization (intercurrent) events that are related to both treatment assignment and outcome, which must be foreseen and addressed in study planning and statistical analysis.^9^, ^10^ It is possible to minimize bias from some postbaseline differences through study protocols requiring the groups to be treated the same, for example, by requiring stability of dose for chronic therapies such as prednisone. However, the study design cannot ensure that there will be no differences between groups in rates of events such as rescue therapy.

Rescue therapy rates vary by study, for example, rescue therapy was required in only 3/200 (1.5%) in the MycarinG study, but 18/125 (14.4%) in the REGAIN study (Table 1).^11^, ^12^ Several factors may account for differences in rescue therapy rates, including trial duration (e.g., McarinG: 6 weeks; REGAIN; 26 weeks) and inclusion criteria or random differences across studies in MGFA class or baseline disease severity (e.g., baseline MG‐ADL at least three in MycarinG; at least six in REGAIN). Restricting enrollment criteria may reduce rescue therapy rates, but arguably at the price of excluding the patients most in need of effective therapies, that is, those with poorly controlled MG. New trials for novel therapies in MG are increasingly focused on treatment‐resistant (refractory) MG, which may lead to increased rescue therapy rates.^13^ Moreover, because it is impossible to know what rescue therapy rates will be prior to conducting the trial and funding agencies require a priori‐specified statistical analysis plans, potential biases introduced by rescue therapy should be addressed during trial planning and design.

Exactly how to incorporate rescue therapy into the study planning and statistical analysis depends on the therapeutic question. For example, if rescue therapy is viewed as an endpoint in addition to functional status, then the question is “What is the effect of treatment on how patients function or feel, and the risk of requiring rescue therapy?” That is, an effective treatment would improve daily functioning and/or reduce the risk of requiring rescue therapy. Conversely, the question could focus on the effect of treatment if rescue therapy had not been available (or more generally, if the patient simply did not receive rescue therapy when it was required, whatever the reason for its omission), or perhaps address the effect of treatment prior to requiring rescue therapy. Each of these is essentially a different way of defining the treatment effect, and thus answers to each of these questions may differ from each other.^14^ They also answer fundamentally distinct, if related, therapeutic questions whose relevance to patients and physicians may vary by circumstance. Moreover, appropriate statistical analyses vary by how the treatment effect is defined, and some statistical analyses implicitly correspond to a specific definition, suggesting that at least some questions/disagreements about the optimal statistical analysis are in part questions/disagreements about what treatment effect definition is most appropriate for a given trial.^10^ That is, the treatment effect definition should be determined *prior* to the statistical analysis plan, not vice versa.

In what follows we discuss strategies for incorporating rescue therapy in the design and statistical analysis of MG trials, show how each strategy affects the interpretation of trial results, and suggest circumstances when each strategy may or may not be applicable to patients or physicians. Regulatory agencies are increasingly concerned that trials are clear about how intercurrent events such as rescue therapy are addressed because clear plans for their handling can reduce the risk of bias. Since the estimand framework is well suited to such considerations and recommended by regulatory agencies,^10^, ^15^, ^16^, ^17^ our discussion will be filtered through this lens.

## Estimand Framework

An *estimand* precisely defines the treatment effect to be estimated by the study, including as part of its definition (1) the population of interest to whom the study results should generalize, for example, adults with refractory MG, (2) the endpoint obtained for each patient, for example, change‐from‐baseline to week 12 in MG‐ADL, (3) the treatment regimens, for example, a well‐defined dose schedule for eculizumab versus placebo, (4) the population‐level summary statistic for comparing the endpoints between treatment regimens, for example, the difference in means, and (5) the strategy for addressing postrandomization (intercurrent) events, the latter of which is often neglected and is the focus of the present work.^10^, ^15^ The estimand is therefore the quantity we need to estimate to answer the therapeutic question.

ICH guidelines specify five primary strategies for handling intercurrent events, each of which we will apply in the context of rescue therapy for MG trials. (1) *Treatment policy* strategies ignore the occurrence of rescue therapy and thus include each patient's data in the analysis in the same manner regardless of the occurrence of rescue therapy. (2) *Hypothetical* strategies attempt to estimate the treatment effect under the scenario that rescue therapy was not administered for any patients. (3) *While‐on‐treatment* strategies typically describe treatment comparisons prior to the occurrence of rescue therapy. (4) *Composite* strategies expand the definition of the endpoint to encompass rescue therapy in addition to the functional endpoint. (5) *Principal stratum* strategies in their simplest form attempt to define a subpopulation in which (a) rescue therapy would not have been required regardless of treatment assignment or (b) rescue therapy would have been required regardless of treatment assignment and estimate the treatment effect within that subpopulation alone. In the following section, we elaborate on each of these strategies for addressing rescue therapy in MG trials. See Table 2 for a summary of strategies employed in recent MG trials.

**Table 2:** Summary of strategies for addressing rescue therapy in MG clinical trials.

	Example therapeutic question	Benefits	Disadvantages
Treatment policy	In patients with MG, what is the mean difference in change‐from‐baseline in MG‐ADL at 12 weeks between patients who take treatment plus (possibly) rescue therapy versus those who take placebo plus (possibly) rescue therapy?	Simpler statistical analyses May be appropriate when rescue therapy is not expected to significantly impact study endpoint Treatment effect (often) in units of functional measure	Ignores rescue therapy in analysis and interpretation Cannot tease apart differences between effects of treatment versus effects of rescue therapy Answers a potentially irrelevant question Increased risk of false positives or bias, decreased power
Hypothetical	In patients with MG, what is the mean difference in change‐from‐baseline in MG‐ADL at 12 weeks between patients who take treatment versus those who take placebo, if no patients receive rescue therapy (or if rescue therapy is not available)?	Isolates the effects of treatment from the effects of rescue therapy Treatment effect (often) in units of functional measure High power and reasonable clinical interpretability when rescue therapy rates are low	Clinical interpretation is complicated/difficult, especially if rescue therapy rates are high Strong assumptions about postrescue therapy functional status for many applicable statistical methods
While‐on‐treatment	In patients with MG, what is the mean difference in change‐from‐baseline in MG‐ADL at 12 weeks or prior to rescue therapy (whichever occurs first) between patients who take treatment versus those who take placebo?	Clinical interpretation is usually straightforward. Treatment effect (often) in units of functional measure Explicitly describes how rescue therapy affects the interpretation of treatment effect	Assumes (precise) duration of treatment is not very important. Requires assumptions about functional status trajectory over time
Composite	In patients with MG, what is the probability that a patient on treatment has either (1) greater improvement in MG‐ADL and/or (2) longer time‐to‐rescue therapy than a patient on placebo after 12 weeks?	Clinical interpretation is usually straightforward Explicitly describes how rescue therapy affects the interpretation of treatment effect Less assumptions are required for many commonly applied statistical methods	Treatment effect is not in units of functional measure Difficulty in determining how much of treatment effect was due to improving MG‐ADL versus reducing risk of rescue therapy Lower power when low rescue therapy rates or only one MG‐ADL or rescue therapy is affected by treatment
Principal stratum	In patients with MG *who would not require rescue therapy, regardless of treatment assignment*, what is the mean difference in change‐from‐baseline in MG‐ADL at 12 weeks between patients who take treatment versus those who take placebo?	Treatment effect (often) in units of functional measure Allows for the defining subpopulations who may respond differently to treatment	Treatment effect may not be applicable to the original population of interest Clinical interpretation is complicated/difficult, especially if rescue therapy rates are high Impossible to know which patients belong to each stratum Strong assumptions required for most applicable statistical methods

## Strategies to Address Rescue Therapy in Treatment Effect Definitions

### Treatment policy

Treatment policy strategies are closely aligned with the intention‐to‐treat (ITT) principle, that is, including all individuals randomized and analyzing the data with respect to randomization not the actual treatment received. In the context of MG trials and rescue therapy, this strategy implies that for the purposes of statistical analysis, we ignore both whether and/or when an individual receives (i) rescue therapy and/or (ii) discontinues treatment (possibly due to having received rescue therapy). Consequently, functional status measures occurring after receipt of rescue therapy would be included in all statistical analyses without any adjustment, statistical or otherwise. As such, a naïve ITT approach in cases where rescue therapy may occur implicitly includes the possibility of receiving rescue therapy as part of each treatment policy. For example, in a trial with eculizumab as the active treatment versus placebo, the treatment regimens are in fact eculizumab plus rescue therapy versus placebo plus rescue therapy. This mirrors the example from the introduction, that is, we cannot disentangle the effects of eculizumab from the effects of rescue therapy and there will be bias toward the null for superiority trials and toward noninferiority for noninferiority trials because rescue therapy tends to make the placebo group appear more similar to experimental treatment group than would be expected in the absence of rescue therapy (assuming an effective treatment).^10^ Thus, the problem is not necessarily that the treatment policy strategy is invalid in principle, but that it may answer an irrelevant question, especially if rates of rescue therapy differ between treatment groups. That is, clinical interest would likely be in a direct comparison of eculizumab versus placebo rather than eculizumab plus rescue therapy versus placebo plus rescue therapy, making the treatment policy strategy less likely to be of primary interest in MG trials. Possible exceptions would be scenarios in which rescue therapy was not expected to have a substantial impact on the endpoint, for example, if follow‐up was long enough such that rescue therapy midway through the study would not be expected to impact MG‐ADL at study end, or rescue therapy is expected to provide only limited relief of MG symptoms.^10^

### Hypothetical

Hypothetical strategies envision circumstances in which, often contrary to fact, rescue therapy was not provided to any patients. For a hypothetical strategy to be valid, it is important to be explicit about the hypothetical scenario one seeks to emulate; otherwise, the interpretation of the results may not have clinical relevance.^10^, ^15^ Hypothetical scenarios often describe circumstances where rescue therapy is either (a) unavailable or (b) not taken by some patients even if available; it is debatable whether either of these scenarios is realistic for rescue therapy in MG patients. However, hypothetical strategies may be easily interpreted by patients and physicians – unlike treatment policy strategies, they explicitly separate the effects of the experimental treatment from rescue therapy, answering therapeutic questions such as “What is the effect on change‐from‐baseline in MG‐ADL if no patients take rescue therapy?” In contrast to composite strategies, the final estimates of treatment effect will often be in the units of functional endpoint, for example, MG‐ADL, so that patients and physicians can easily interpret the level of benefit a treatment confers on average. Note that hypothetical strategies may be significantly more controversial for other postrandomization events, for example, death.^14^

A wide variety of statistical methods is available for hypothetical strategies. The simplest approach is a complete‐case analysis, including in the analysis only individuals who completed the study without requiring rescue therapy, which produces unbiased estimates of the treatment effect only if (a) patients who receive rescue therapy are similar to patients who do not receive rescue therapy and (b) functional endpoints would have been similar in both those who did and did not receive rescue therapy, neither of which is a reasonable assumption in MG trials. Other approaches usually exclude from analysis functional measurements occurring postrescue therapy and either impute or model outcomes that would have occurred if rescue therapy had not been administered.

Simple imputation approaches include last observation carried forward (LOCF) and worse‐score imputation. LOCF makes the strong assumption that if the individual had not received rescue medication, then their remaining functional measurements would not change from what they were prior to rescue medication during the rest of the study, whereas worse‐score imputation (WSI) assumes that those receiving rescue therapy would have functional measurements equal to the worst functional measurements observed in those who did not receive rescue medication, neither of which are reasonable assumptions in most scenarios. More complex models, for example, linear mixed models, multiple imputation, or inverse probability weighting overcome some limitations of LOCF and WSI. The medical statistics and trials literature favors the so‐called mixed model for repeated measures (MMRM), which treats time as a categorical variable, for example, modeling the functional measure at each follow‐up visit rather than smoothing over the entire duration of the study.^10^, ^14^, ^18^ All the aforementioned models assume that the probability of rescue therapy, and hence of missing functional measurements, is not related to the functional measurements that would have occurred in the absence of rescue therapy (missing‐at‐random, MAR), which is unlikely to be a reasonable assumption in the case of rescue therapy (missing‐not‐at‐random, MNAR).^19^ Joint models of longitudinal and time‐to‐event data (also called shared‐parameter models) are in part designed to handle MNAR missingness and therefore can be used to estimate, for example, change‐from‐baseline in MG‐ADL while accounting for the MNAR missingness arising due to rescue therapy.^19^

### While‐on‐treatment

While‐on‐treatment strategies focus on treatment comparisons prior to the occurrence of rescue therapy, ignoring as irrelevant the measurements that occur afterward. Historically, this strategy has been viewed as useful in the case of severe events such as death, answering reasonable questions such as “How much improvement in MG‐ADL can a patient expect after 6‐months or prior to death, whichever happens first?” In such cases, a patient's functional measurements up to the point of death may still be clinically meaningful, and it may strain credulity to posit functional measurements that would have occurred if the patient survived (hypothetical strategy).^10^, ^14^ Implicit in while‐on‐treatment strategies is that the duration of treatment is not particularly important, for example, because change from baseline in MG‐ADL may be based on 3 months of follow‐up for one patient and 6 months of follow‐up for another, etc., depending on when or if the event occurs. This means that the treatment effect is no longer tied to a landmark timepoint, for example, change from baseline in MG‐ADL over 6 months, as is the case in hypothetical strategies. If the duration of experimental treatment is of key clinical relevance, then while‐on‐treatment strategies may not be appropriate.

A simple implementation of a while‐on‐treatment strategy would calculate change‐from‐baseline in MG‐ADL by using only baseline and the last MG‐ADL measurement occurring prior to rescue therapy. This approach is similar to LOCF, except that the interpretation of the study results changes, that is, in LOCF for a hypothetical strategy, we assume that the treatment effect estimate describes the effect of treatment taken over the entire duration of the study, whereas in “while‐on‐treatment,” we assume no particular timeframe for treatment duration, except that it is equal to or less than the follow‐up duration. Linear mixed models that treat time as continuous may also be employed, and unlike MMRM, simultaneously address hypothetical and while‐on‐treatment estimands, although they involve more assumptions about the trajectory of the functional measure over time, and to be validly applied to hypothetical strategies must meet additional assumptions discussed above.^10^, ^14^

### Composite

Composite strategies expand the definition of the endpoint to encompass rescue therapy, in addition to the functional outcome. This implicitly assumes that whether rescue therapy was required is equally as indicative of treatment success/failure as the functional measure. That is, those patients requiring rescue therapy are classified as treatment failures. In the context of MG trials, requiring rescue therapy implies a marked decline in the functional measure, which arguably implies treatment failure since the patient's symptoms had to be severe before rescue therapy was administered. Exceptions may include scenarios where rescue therapy may be required in a timeframe prior to when the experimental treatment is expected to take effect.

While composite endpoints for several binary outcomes can be straightforward to define, for example, a “good” outcome only if none of the variables making up the composite endpoint occur, the situation is more complicated in MG trials because the functional endpoint is a continuous measure. In other contexts, for example, when the intercurrent event is death rather than rescue therapy, it may be acceptable to assign the worst possible score to patients who experience the event.^10^, ^14^ However, this approach suffers from the fact that (1) it is often unreasonable to assume that, for example, receiving rescue therapy is equivalent to the worst possible MG‐ADL score, especially if no other patients in the study have so severe a score and (2) it ignores the time at which rescue therapy occurred, which is relevant because presumably patients who require rescue therapy earlier in the trial should be considered as having worse outcomes than patients who receive rescue therapy later in the trial. In contrast, ranked‐based approaches can combine time‐to‐rescue therapy and functional endpoint data by ranking the patient who first requires rescue therapy last, followed by the next patient to require rescue therapy, and so forth, after which patients who did not require rescue therapy are ranked by their functional endpoint measures.^10^, ^14^, ^20^ The corresponding statistical hypothesis test is based on a Wilcoxon rank‐sum test, reduces the potential for tied observations, and has superior statistical power.^14^, ^21^ The downside to this approach is the lack of interpretability, for example, the treatment effect is a comparison of the sum of ranks in each group, which is not clinically interpretable, much less interpretable in the original units of a functional score such as MG‐ADL.^10^, ^12^, ^14^

Several extensions of the rank‐based approach improve clinical interpretability. The win‐ratio approach compares each patient's endpoint to every other patient's endpoint, in each case determining which patient had the better (win) and worse (loss) outcome; the win‐ratio is then the number of wins divided by the number of losses in the experimental treatment group, that is, the odds of having a better outcome on the experimental treatment versus the control^22^, ^23^, ^24^; *p*‐values and confidence intervals are straightforward to obtain. A similar ranked‐based method is the desirability of outcome ranking (DOOR), whose treatment effect estimate is the probability that a randomly selected patient would have a better outcome on, for example, eculizumab versus placebo; DOOR also produces *p*‐values and confidence intervals.^25^

A remaining consideration for rank‐based approaches is whether the assumption that receiving rescue therapy is inherently worse than any possible or observed functional score is reasonable. A final analytic option that does not necessarily assume primacy of the time‐to‐rescue therapy or change in the functional measure is a joint model of longitudinal and survival outcomes (JM), for example, by modeling MG‐ADL with a linear mixed model to obtain an estimate of change‐from‐baseline and modeling time‐to‐rescue therapy with a proportional hazards model, but estimating the two components within a single framework to account for dependence between the two outcomes.^19^ An underappreciated benefit of these models is that they can provide estimates of the treatment effect with respect to (1) change‐from‐baseline in MG‐ADL, (2) time‐to‐rescue therapy, and (3) an “overall” treatment effect that is a combination of the first two treatment effects.^26^, ^27^ Note that if there are very few instances of rescue therapy, then the time‐to‐event submodel may be unstable or unreliable, which is not an issue with rank‐based approaches.

### Principal stratum

Principal stratum strategies assume that patients can be divided into subpopulations based on whether they would require rescue therapy, that is, individuals who would (a) not require rescue therapy regardless of treatment assigned, (b) require rescue therapy regardless of treatment assigned, (c) require rescue therapy if assigned to active treatment but not if assigned to the control treatment, and (d) require rescue therapy if assigned to the control treatment but not if assigned to the active treatment. It is then possible to estimate causal effects within each stratum/subpopulation, and the target population of interest is usually those who would never require rescue therapy (that is, case (a)).^10^, ^15^

In practice, it is not possible to know to which stratum a patient belongs, although we may be able to know (after the study is completed) that a patient is definitely not in a particular stratum. For example, if a patient in the control group does not require rescue therapy, then we know that they cannot be from stratums (b) or (d), but we cannot be certain whether they belong to stratum (a) or stratum (c) because they were not observed under the experimental treatment. Thus, principal stratum strategies differ from subgroup analyses because stratum membership must be estimated/predicted based on available data. Therefore, the validity of the results depends on the accuracy of the model used to predict stratum membership, which in turn can depend on which covariates are measured and the sample size of the study. Principal stratum strategies may also shift the population of interest, for example, from a broader population of MG patients to those patients who would never require rescue therapy, and only those predicted to be members of the strata are used for analysis, not all patients enrolled in the trial. However, average treatment effects in the overall population may be estimated as weighted averages of the treatment effects within each stratum, but it is debatable whether the additional complexity is justified if the treatment effects within some or all strata are not of clinical interest, and principal stratum strategies have thus far not been widely applied in practice.^14^, ^28^ Especially in the case of refractory MG, it is not clear that the subpopulation that would *never* require rescue therapy is the population of interest, since conceivably those patients may already have well‐controlled MG. Moreover, we may doubt whether commonly defined principal strata correspond to well‐defined subpopulations, for example, perhaps patients have varying levels of probability of requiring rescue therapy under a particular treatment, not a fixed‐in‐advance outcome.

## Conclusion

We have reviewed available strategies for addressing rescue therapy in MG trials and endeavored to show why doing so is important for clinical interpretation and validity. Imprecise or haphazard approaches to addressing rescue therapy, including outright ignoring rescue therapy, can produce biased results or answer a therapeutic question that is irrelevant or different from the one intended. To avoid such pitfalls, we recommend first using the strategies presented to align the therapeutic question with the treatment effect definition (estimand) during study planning, after which the statistical analysis plan should be designed around that definition; it is inadvisable to allow the statistical analysis plan to implicitly define the treatment effect. Moreover, clearly specifying how rescue therapy will be addressed a priori will not only improve the robustness of the study but also increase alignment with expectations/recommendations of regulatory and/or funding agencies.

## Author Contributions

Justin M. Leach: conceptualization, literature review, writing – original draft, writing – review and editing. Inmaculada Aban: conceptualization, writing – review and editing. Michael Benatar: conceptualization, writing – review and editing. Gary Cutter: conceptualization, writing – review and editing.

## Conflict of Interest

Justin M. Leach reports no conflicts of interest. Michael Benatar reports research support from Alexion and Immunovant and has served as a paid scientific consultant to Alexion, Amgen, Canopy, Cartesian, CorEvitas, Immunovant, and Sanofi. Inmaculada Aban reports research support from Verona, Myasthenia Gravis Foundation of America (MGFA), Ra/UCB through MGFA; paid scientific consultant to Roche.

## Data Availability

R code used to simulate the data for Figure 1 is publicly available at https://github.com/jmleach‐bst/Rescue‐Therapy‐MG‐Review.
